# Poor long-term outcomes and abnormal neurodegeneration biomarkers after military traumatic brain injury: the ADVANCE study

**DOI:** 10.1136/jnnp-2024-333777

**Published:** 2024-10-11

**Authors:** Neil SN Graham, Grace Blissitt, Karl Zimmerman, Lydia Orton, Daniel Friedland, Emma Coady, Rhiannon Laban, Elena Veleva, Amanda J Heslegrave, Henrik Zetterberg, Susie Schofield, Nicola T Fear, Christopher J Boos, Anthony M J Bull, Alexander Bennett, David J Sharp, for the ADVANCE Study

**Affiliations:** 1 Department of Brain Sciences, Imperial College London, London, UK; 2 UK Dementia Research Institute Centre for Care Research and Technology, London, UK; 3 Academic Department of Military Rehabilitation, Defence Medical Rehabilitation Centre, Loughborough, UK; 4 Department of Bioengineering, Imperial College London, London, UK; 5 UK Dementia Research Institute at UCL, London, UK; 6 Department of Neurodegenerative Disease, UCL Queen Square Institute of Neurology, London, UK; 7 Institute of Neuroscience and Physiology, Goteborgs Universitet, Goteborg, Sweden; 8 National Heart and Lung Institute, Imperial College London, London, UK; 9 King's Centre for Military Health Research, King's College London, London, UK; 10 Academic Department for Military Mental Health, King's College London, London, UK; 11 Faculty of Health & Social Sciences, Bournemouth University, Poole, UK; 12 Centre for Injury Studies, Imperial College London, London, UK; 13 Care Research & Technology, UK Dementia Research Institute, London, UK

**Keywords:** dementia, traumatic brain injury, head injury

## Abstract

**Background:**

Traumatic brain injury (TBI) is common in military campaigns and is a risk factor for dementia. *A*rme*D* Ser*V*ices Tr*A*uma and Rehabilitatio*N* Out*C*om*E*-TBI (ADVANCE-TBI) aims to ascertain neurological outcomes in UK military personnel with major battlefield trauma, leveraging advances in quantification of axonal breakdown markers like neurofilament light (NfL), and astroglial marker glial fibrillar acidic protein (GFAP) in blood. We aimed to describe the causes, prevalence and consequences of TBI, and its fluid biomarker associations.

**Methods:**

TBI history was ascertained in 1145 servicemen and veterans, of whom 579 had been exposed to major trauma. Functional and mental health assessments were administered, and blood samples were collected approximately 8 years postinjury, with plasma biomarkers quantified (n=1125) for NfL, GFAP, total tau, phospho-tau_181_, amyloid-β 42 and 40. Outcomes were related to neurotrauma exposure.

**Results:**

TBI was present in 16.9% (n=98) of exposed participants, with 46.9% classified as mild-probable and 53.1% classified as moderate to severe. Depression (β=1.65, 95% CI (1.33 to 2.03)), anxiety (β=1.65 (1.34 to 2.03)) and post-traumatic stress disorder (β=1.30 (1.19 to 1.41)) symptoms were more common after TBI, alongside poorer 6 minute walk distance (β=0.79 (0.74 to 0.84)) and quality of life (β=1.27 (1.19 to 1.36), all p<0.001). Plasma GFAP was 11% (95% CI 2 to 21) higher post-TBI (p=0.013), with greater concentrations in moderate-to-severe injuries (47% higher than mild-probable (95% CI 20% to 82%, p<0.001). Unemployment was more common among those with elevated GFAP levels post-TBI, showing a 1.14-fold increase (95% CI 1.03 to 1.27, p<0.001) for every doubling in GFAP concentration.

**Conclusions:**

TBI affected nearly a fifth of trauma-exposed personnel, related to worse mental health, motor and functional outcomes, as well as elevated plasma GFAP levels 8 years post-injury. This was absent after extracranial trauma, and showed a dose-response relationship with the severity of the injury.

**WHAT IS ALREADY KNOWN ON THIS TOPIC**

- Military neurotrauma has been associated with increased dementia risk, but a detailed understanding of the long-term neurological effects of battlefield injuries has been lacking, with ultrasensitive neurodegeneration markers not been explored in a midlife cohort such as the *A*rme*D* Ser*V*ices Tr*A*uma and Rehabilitatio*N* Out*C*om*E* study (ADVANCE).

**WHAT THIS STUDY ADDS**

- We have shown that traumatic brain injury (TBI) is highly prevalent in the cohort (around a fifth), and that the astroglial activation marker glial fibrillar acidic protein (GFAP) is abnormally elevated in the blood an average of 8 years post-injury, associated with worse employment outcomes.

**HOW THIS STUDY MIGHT AFFECT RESEARCH, PRACTICE OR POLICY**

- TBI outcomes are heterogeneous, with a significant proportion of patients experiencing deterioration late postinjury.
- Identifying people at highest risk of progressive problems is important for future treatment trials, and may inform clinical monitoring and care in the interim.

## Introduction

 Traumatic brain injury (TBI) is a common consequence of warfare, and was particularly frequent during the Afghanistan campaign from 2002 to 2014, partly due to the use of improvised explosive devices.^1 2^ Neurodegenerative diseases such as Alzheimer’s disease (AD), Parkinson’s disease and chronic traumatic encephalopathy (CTE) are seen more commonly after TBI, a particular concern in military settings where there is a risk of occupational trauma, including blast injury.^3^,^5^ Dementia risk appears to increase in a dose-response manner with the severity and number of injuries.^6^

Clinical outcomes more broadly after TBI are increasingly recognised as dynamic over time, with many patients improving but a significant proportion worsening several years postinjury.^7^,^9^ Cognitive problems due to axonal damage and brain network dysfunction,^10^ and psychiatric symptoms, such as depression, anxiety and post-traumatic stress disorder (PTSD) are an important contributor to postinjury disability.^11^ However, the extent to which these reflect variability around a new postinjury baseline, versus symptoms of a progressive problem, such as traumatic encephalopathy syndrome, remains hard to assess.^12^

Recent advances in analytic techniques allow for sensitive quantification of neurodegeneration-associated proteins in peripheral blood, aiding in the assessment of acute injury as well as the characterisation of chronic postinjury neurodegeneration.^13^ These markers are increasingly close to integration into clinical pathways in AD: for example, early reductions in the amyloid-β (Aβ) 42:40 ratio and elevation in phospho-tau species (eg, phosphorylated at serine 181, p-tau_181_ and tau phosphorylated at threonine 217, p-tau_217_) are sensitive to amyloid pathology.^14 15^ Neurodegeneration may be indexed by increased levels of total tau, or the axonal marker plasma neurofilament light (NfL). Furthermore, associated processes may be measured, for example, astroglial activation, quantified by marker glial fibrillar acidic protein (GFAP), which is elevated in plasma early in AD.^16^

In moderate-to-severe TBI, the neuro-axonal degeneration marker NfL increases rapidly and remains elevated a year later.^13^ Ongoing elevation has been reported as long as 5^17^ to 15 years post-TBI,^18^ but investigations into this late stage are limited.^19^ Interestingly, raised plasma NfL in the chronic phase years postinjury may predict progressive deterioration in functional status.^19^ The astroglial marker GFAP has been also shown to be elevated in plasma 1–5 years post-injury .^13 17^ While neuronal markers such as total tau, ubiquitin carboxyl hydrolase L1 are elevated acutely and normalise within days of injury,^13^ phospho-tau_181_ (considered a marker of amyloid-driven tau pathology, correlating with both amyloid plaque and neurofibrillary tangle burden^20 21^) is not raised in the first year postinjury.^22^

The *A*rme*D* Ser*V*ices Tr*A*uma and Rehabilitatio*N* Out*C*om*E* study (ADVANCE)-TBI study^23^ was established to clarify the long-term consequences of military TBI within the large (n=1145) UK cohort study of military personnel who served in Afghanistan.^24^ Our aims here were to describe the prevalence and types of TBI in the cohort and to report on the influence of TBI on neurodegeneration biomarkers, as well as psychiatric and functional outcomes. Specifically, we hypothesised that: (1) plasma concentrations of NfL and GFAP would be significantly increased after TBI; (2) that combat TBI would be associated with worse psychiatric (anxiety, depressive and PTSD symptoms) and functional outcomes (employment status, 6 min walk distance^25^ and quality of life) and (3) that such outcomes and plasma concentrations of NfL and GFAP would be associated in patients after TBI.

## Materials and methods

### Study population

1145 combat personnel were assessed in the ADVANCE cohort study baseline visit. All participants were eligible for inclusion in this substudy of TBI outcomes (‘ADVANCE-TBI’).^23^ As previously described,^24^ all participants were recruited using Defence Statistics UK data covering serving and ex-serving military personnel. This comprised a list of military personnel who sustained a combat injury (n=1400) and a separate list of men who had not sustained an injury. Combat trauma-exposed participants (n=579) were recruited, along with an uninjured comparison group (n=566) deployed during the same period, which was frequency-matched for age, service, role, time of deployment and regiment. Te exposed and unexposed groups were well-matched with respect to age and ethnicity. Sample size for the cohort was previously determined.

Overall injury severity was quantified using the New Injury Severity Score (NISS).^26^ Depressive symptoms were measured using the Patient Health Questionnaire-9 (PHQ9, possible range 0–27), anxiety symptoms were assessed with the Generalised Anxiety Disorder-7 (GAD7, range 0–21) and PTSD symptoms were evaluated using the PTSD Checklist for Diagnostic and Statistical Manual of Mental Disorders, fourth edition (PCL4, range 17–85). Quality of life was indexed using the EuroQol EQ-5D-5L instrument.^27^ Standardised cut-offs were used on the psychiatric symptom questionnaires to denote clinically meaningful symptom burden. A clinical cut-off of ≥50 was used to define significant PTSD symptoms on the PCL4 instrument, whereas a cut-off of ≥5 was used on both of the GAD7 anxiety and PHQ9 depression scoring tools (indicating at least clinically mild symptoms for these measures).

### TBI ascertainment and definition

In the ADVANCE study, combat trauma-exposed participants were defined as those with exposure to major battlefield trauma requiring aeromedical evacuation back to the UK with hospital admission, but not necessarily TBI. To establish the presence or absence of TBI, medical history data, collected using study case report forms and trauma registry data (joint theatre trauma registry), were reviewed by two of the study investigators, including a neurologist. Clinical features, including relevant imaging findings, neuropathologies, lowest conscious level, duration of post-traumatic amnesia, and other available details, were documented.

The Mayo classification was used to separate injuries into moderate-to-severe (definite), mild (probable) and symptomatic (possible), using information spanning several domains, such as clinical features (eg, lowest Glasgow Coma Scale (GCS)) and neuroimaging (CT or MRI findings).^28^ The Mayo classification shares similarities with other classification tools, including the Veterans Affairs (VA) / Department of Defense (DOD) TBI definition/severity schema.^29^ The most notable contrast is that moderate and severe categories are combined in the Mayo classification. Mayo injury severities are accompanied by a confidence level, such as possible, probable or definite. Injuries may be classified as moderate-to-severe (definite) in the Mayo classification if the structural neuroimaging is abnormal (other than skull fracture), similarly to moderate and severe groups in the VA/DoD schema. Likewise, loss of consciousness of >30 min prompts classification as moderate-to-severe in the Mayo classification, as this does for moderate in the VA/DoD schema. Post traumatic amnesia (PTA) of >24 is sufficient for moderate-to-severe classification in the Mayo system, as in the VA/DoD moderate category. A lowest GCS of 12 and below defines Mayo moderate-to-severe injuries, with the same threshold used for moderate (9–12) in the VA/DoD.

Mayo mild (probable) cases are defined by any loss of consciousness from momentary to 30 min, PTA <24 hours or GCS 13–15 as per the VA/DoD mild cases; however, any depressed, basilar or linear skull fracture (dura intact) would also lead to a mild injury classification in the Mayo system (irrespective of other features). The Mayo classification includes a further mildest category termed ‘symptomatic possible’, which would include hits to the head with symptoms such as mental status change, dizziness, headache or nausea. Since these features provide a much lower level of certainty, they have not been included in our case definition of TBI for this investigation.

### Fluid biomarkers

Peripheral venous blood was sampled and collected using EDTA tubes, stored for 30 min at room temperature and centrifuged at 4°C at ~1750 g for 20 min at the baseline assessment within the ADVANCE study. Samples were aliquoted and frozen at −80°C. A bead-based digital enzyme-linked sandwich immunoassay was performed at the UK DRI Biomarker Factory at UCL to quantify plasma biomarkers using a HD-X platform, following the manufacturer’s instructions (Quanterix).^13^ Aβ42, Aβ40, GFAP and NfL concentrations were assessed in plasma using a Neurology 4-plex E assay. Thawing was performed at room temperature, followed by centrifugation (10 000×g for 5 min, room temperature). A 1:4 dilution was used to measure plasma samples, on board the instrument. Control samples with high and low concentrations of the proteins of interest were run for quality control. Four internal controls made of pooled plasma were included on every plate to monitor intraplate coefficient of variation % (CV%) and interplate CV%. A calibration curve was generated (four parameter logistic curve fit data reduction method). Samples were run in duplicate, and the mean used in further analyses. The lower limits of quantification were 11.6 pg/mL for GFAP, 1.6 pg/mL for NfL, 4.08 pg/mL for Aβ42, 1.51 pg/mL for Aβ40, 0.248 pg/mL for t-tau and 0.338 pg/mL for p-tau_181_. Results falling below the lower limit of quantification (LLQ) were recorded as 50% of the LLQ (this was performed for two NfL results, four Aβ42 results, one GFAP result and three tau results). All interplate CV%s were below 15%. All intraplate CV%s were below 20%, of which all but two were below 15% (both 16.5%).

### Statistical analyses

Due to the skewed distribution of fluid biomarker results, the data were log transformed (using natural logarithms; other than the logistic regression relating GFAP and employment outcomes, where a base-2 log was used, for ease of interpretation) and these data are presented as geometric means and geometric SD. A complete cases approach was employed to address to missing data. Information on missing values is provided in each table, as well as in the online supplemental file.

Linear regressions were used to test the association between TBI and fluid biomarker concentrations, psychiatric symptom scales, total quality of life scores and 6 min walk distance. In linear models, the fluid biomarkers were log transformed and geometric means ratios and 95% CIs were reported. To investigate the association between these measures and injury exposure, log-transformation was also performed for the outcomes GAD7, PHQ9, PCL4, EQ-5D-5L and 6 min walk scores (due to non-linearity). In order to log transform PHQ9 and GAD7, a constant of 1 was added to the score due to the existence of scores of 0. Logistic regression was used for the employment (binary in vs out of work/training) outcome. Military rank at the time of deployment/injury, as a surrogate of socioeconomic status, and age were both included a priori as confounders in all models. In models involving walking speed, amputee status was included as an additional confounder given its likely substantial influence on physical mobility.

Three groups were defined across the ADVANCE cohort and used in these analyses: unexposed comparison group (‘uninjured’), participants exposed to extracranial injuries but not TBI (‘extracranial injury’ group) and participants with TBI. In the first stage of each analysis, all participants are included, and those with TBI are compared with the reference group of unexposed controls. To confirm that associations are specific to TBI rather than trauma in general, a post hoc test (Tukey’s test, including multiple comparison correction) was done to compare TBI with extracranial injury.

Individuals with ‘abnormally elevated’ GFAP were identified by taking the unexposed participant distribution of that marker and generating a cut-off at the 97.5th centile. Exploratory comparisons of patients with TBI with raised versus non-raised levels were conducted. The Wilcoxon test was used to compare the injury severity score and time since injury. A further exploratory analysis of the influence of time since injury on fluid biomarker concentration was undertaken. Specifically, within the exposed group only, an interaction was tested between the presence of TBI and years since trauma exposure, while accounting for age/rank as confounders. Main effects of TBI presence/time since injury were also included in the analysis.

The association between fluid biomarkers of injury/neurodegeneration and clinical outcomes (ie, function/psychiatric measures) was tested in patients after TBI using Spearman’s correlation (continuous variables), or logistic regression (in the case of the binary employment outcome). On the regression, outcome measure of interest was the dependent variable and log-transformed biomarker concentration the independent variable, with age and rank included as confounders. R V.4.3.1 was used.^30^

## Results

### Demographics and injury characteristics

Across the entire cohort of 1145, study participants were 34.1 years of age (mean; SD 5.38) when assessed (table 1). Assessments were typically 8.3 years (mean, SD 2.1) following major combat trauma in the trauma-exposed group. TBI, classified as mild-probable or moderate-to-severe according to the Mayo classification, was identified in 16.9% (n=98) of the trauma group. Of these cases, 46.9% (n=46) were classified as moderate-to-severe and 53.1% (n=52) as mild-probable. Trauma registry data were available for 91 (92.9%) participants in the TBI group, being unavailable in 8 mild-probable but no moderate-to-severe cases. Most mild TBI diagnoses were based on self-reported past medical history, whereas moderate-to-severe cases were more frequently classified using registry data, such as CT findings. The most common CT abnormality in the TBI group was contusions, in 18.4% (n=18) of cases, followed by subarachnoid haemorrhage in 9.2% (n=9), subdural haematoma in 10.2% (n=10), extradural haematoma in 4.1% (n=4) and diffuse axonal injury in 4.1% (n=4). Blast injuries, comprising mixed primary, secondary and tertiary injury mechanisms, were more commonly identified as the cause of moderate-to-severe TBI (82.6%, n=38) compared with mild-probable TBI (78.8%, n=41).

**Table 1:** Demographics and trauma exposure in the ADVANCE cohort

		Unexposed	Major combat trauma exposed			
			Extracranial injuries	TBI		
				All TBIs	Mild-probable subgroup	Moderate-to-severe subgroup
n		566	481	98	52	46
Age at visit (mean (SD))		34.24 (5.41)	33.77 (5.26)	35.19 (5.66)	36.19 (5.54)	34.07 (5.63)
Ethnicity (%)	Caucasian	513 (90.6)	431 (89.6)	93 (94.9)	49 (94.2)	44 (95.7)
	Other	53 (9.4)	50 (10.4)	5 (5.1)	3 (5.8)	2 (4.3)
Injury age (mean (SD))		—	25.52 (5.12)	26.57 (5.26)	27.74 (5.29)	25.26 (4.95)
Rank (%)	Lower rank	270 (47.7)	330 (68.6)	71 (72.4)	34 (65.4)	37 (80.4)
	Mid rank	210 (37.1)	98 (20.4)	19 (19.4)	13 (25.0)	6 (13.0)
	Senior rank	86 (15.2)	53 (11.0)	8 (8.2)	5 (9.6)	3 (6.5)
Amputee (%)		1 (0.2)	138 (28.7)	23 (23.5)	12 (23.1)	11 (23.9)
Trauma mechanism (%)	Unknown	—	33 (6.9)	9 (9.2)	8 (15.4)	1 (2.2)
	Assault	—	0 (0.0)	1 (1.0)	0 (0.0)	1 (2.2)
	Blast	—	323 (67.2)	79 (80.6)	41 (78.8)	38 (82.6)
	Fall	—	0 (0.0)	1 (1.0)	1 (1.9)	0 (0.0)
	Gunshot	—	124 (25.8)	8 (8.2)	2 (3.8)	6 (13.0)
	RTA	—	1 (0.2)	0 (0.0)	0 (0.0)	0 (0.0)
NISS (median (Q25–Q75))		—	12.0 (5.0, 22.0)	16.0 (6.0, 27.0)	12.12 (11.25)	25.70 (13.99)
Required intubation (%)	Yes	—	90 (18.7)	22 (22.4)	6 (11.5)	16 (34.8)
Lowest GCS (%)	Unknown	—	81 (16.8)	29 (29.6)	19 (36.5)	10 (21.7)
	13 to 15	—	330 (82.5)	52 (75.4)	33 (63.5)	19 (41.3)
	9 to 11	—	63 (15.8)	12 (17.4)	0 (0.0)	12 (26.1)
	3 to 8	—	7 (1.8)	5 (7.2)	0 (0.0)	5 (10.9)
LOC (%)*	<30 m	—	—	14 (14.3)	14 (26.9)	0 (0.0)
	>30 m	—	—	2 (3.1)	0 (0.0)	2 (4.3)
CT—skull fracture		—	—	27 (27.6)	5 (9.6)	22 (47.8)
CT—focal pathology	Any focal pathology	—	—	28 (28.6)	0 (0.0)	28 (60.9)
	SDH	—	—	10 (10.2)	0 (0.0)	10 (21.7)
	EDH	—	—	4 (4.1)	0 (0.0)	4 (8.7)
	Contusion	—	—	19 (19.3)	0 (0.0)	19 (41.3)
	SAH	—	—	9 (9.2)	0 (0.0)	9 (19.6)
	DAI	—	—	4 (4.0)	0 (0.0)	4 (8.7)
Neurosurgery (%)		—	—	8 (19.0)	0 (0.0)	8 (17.4)

‘Unknown’ denotes missing or unavailable data, complete data were available for all other variables.

* LOC was self-reported in 13 out of 14 cases in the mild TBI group. Similarly, LOC was self-reported in 1 out of 2 cases in the moderate-to-severe category; however, LOC alone did not determine the moderate-to-severe classification.

ADVANCE*A*rme*D* Ser*V*ices Tr*A*uma and Rehabilitatio*N* Out*C*omEDAIdiffuse axonal injuryEDHextradural haematomaGCSGlasgow Coma ScaleLOCloss of consciousnessNISS, New Injury Severity Score; RTA, road traffic accidentSAHsubarachnoid haemorrhageSDHsubdural haematomaTBItraumatic brain injury

Most trauma was due to blast, comprising 80.6% of the TBI group and 67.2% of the extracranial trauma group. Conversely, the proportion with gunshot trauma was 25.8% in participants with extracranial injury and 8.2% in the TBI group. NISS was 16 (median, Q25–Q75 6–27) in TBI group and 12 (5–22) in participants with extracranial injuries only. Endotracheal intubation for mechanical ventilation was more common after moderate-to-severe TBI (34.8%) than with mild TBI (11.5%) and extracranial injury (18.7%) groups. Neurosurgical procedures such as primary or secondary decompression were performed in eight participants with TBI (17.4% of the moderate-to-severe group, n=8/46), and nil others. There was no significant difference in the number of amputees in the TBI and polytrauma groups (χ^2^=0.86, df=1, p=0.353). Neurological and psychiatric comorbidities are shown in online supplemental table 1, as are non-index TBIs. There were a number of psychiatric disorders reported, most commonly PTSD, depression and anxiety. Seizures were the most common neurological disorder, but there were notably no cases of inflammatory disease or neurodegenerative disease/dementia. Two (0.2%) individuals had moderate-to-severe TBIs separate from the index injury, with both occurring >5 years prior to plasma sampling. Additionally, a total of 62 (5.4%) participants had one or more mild-probable TBI.

### Neuropsychiatric symptoms after TBI

**Figure 1:**
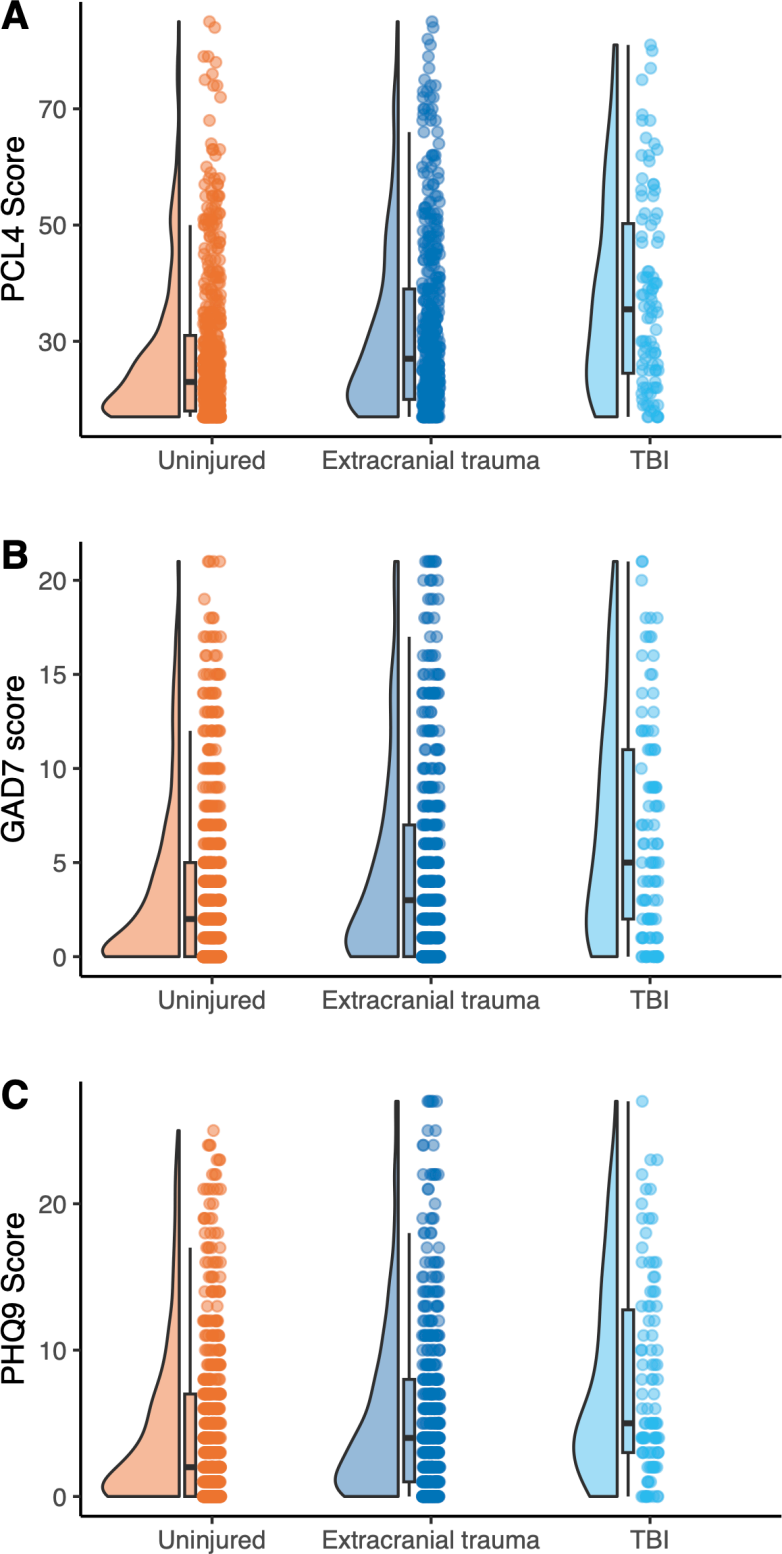
Neuropsychiatric symptoms after traumatic brain injury (TBI). (**A**) Extent of post-traumatic stress disorder (PTSD) symptoms, assessed using the PTSD checklist for Diagnostic and Statistical Manual of Mental Disorders, fourth edition (PCL4) total score, split by study group. (**B**) Anxiety symptoms on the Generalised Anxiety Disorder-7 (GAD7) questionnaire, split by study group. (**C**) Depressive symptoms on Patient Health Questionnaire-9 (PHQ9) instrument by study group. Raincloud plots shown, indicating distribution (left), box plot (middle) and raw data (right) per group, per variable. ‘Uninjured’=uninjured controls shown in orange, ‘extracranial trauma’=participants with extracranial injuries only, but no TBI shown in blue, ‘TBI’=participants with mild-probable or moderate-to-severe TBI, shown in cyan.

The presence of psychiatric symptoms in the ADVANCE cohort was assessed (figure 1, online supplemental table 2). PTSD symptoms were more common in participants after TBI versus uninjured controls, scoring 30% higher on the PCL4 (95% CI 19 to 41, p<0.001, linear regression accounting for age and rank, online supplemental table 2), and this was significant on testing of TBI versus extracranial injury participants (17% higher (95% CI 7 to 27), p=0.001). 25.5% of the TBI group scored in the abnormal range, compared with 9.2% in the uninjured group. Anxiety symptoms were significantly more common after TBI, with GAD7 scores being 65% higher (95% CI 34 to 103, p<0.001) compared with those without TBI. Anxiety symptoms were also higher in the TBI group compared with participants with extracranial injuries, with scores 40% higher (95% CI 14 to 73, p=0.001). In the TBI group, 55.1% of individuals scored above the clinical cut-off for mild or greater anxiety, compared with 29.0% in the uninjured group. Depressive symptoms were also more common after TBI (65% higher (95% CI 34 to 103), p<0.001), significant versus extracranial injury (39% higher (13 to 72), p=0.002), with 56.1% endorsing at least mild depressive symptoms post-TBI, compared with 36.7% in the uninjured group.

### Quality of life, motor function and employment

**Figure 2:**
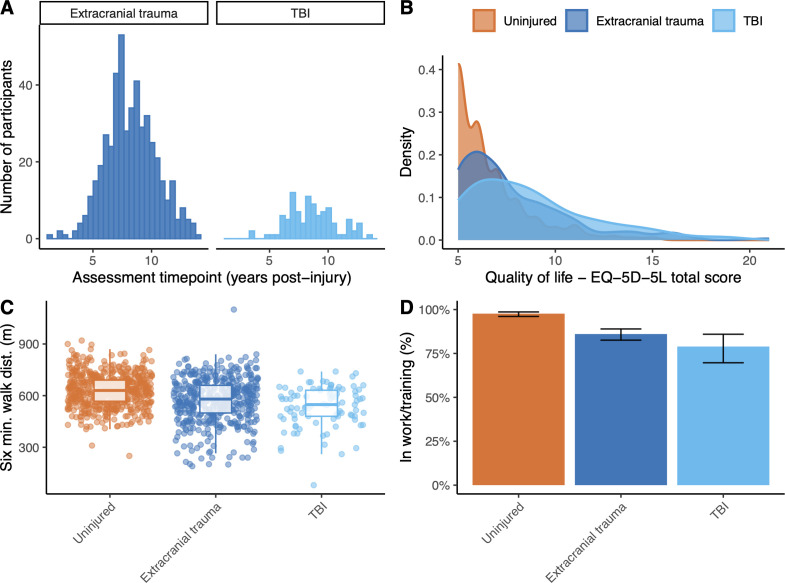
Quality of life, motor function and employment outcomes. (**A**) Assessment timepoint in trauma-exposed group, with count shown against years postinjury for individuals with extracranial trauma (left) and traumatic brain injury (TBI) (right). (**B**) Distribution of total scores on the EQ-5D-5L quality of life self-rating scale, with uninjured participants shown in yellow, participants with extracranial injuries only shown in red and participants with TBI in grey; (**C**) 6 min walk distance and (**D**) employment status by group, with 95% CIs shown for estimate of proportions. Uninjured participants shown in orange, extracranial injuries in blue and TBI in cyan.

TBI was associated with a significantly worse quality of life, with a higher total score on EQ-5D-5L (27% higher (95% CI 19 to 36), p<0.001) than trauma-unexposed participants, a significant difference present on post hoc testing comparing participants with TBI versus those with extracranial injury alone (p=0.010) (figure 2, online supplemental table 3).

Motor function was assessed in the cohort by 6 min walk distance. Participants with TBI covered significantly less distance (0.79 times the uninjured participants (95% CI 0.74 to 0.84), p<0.001), which on testing TBI versus extracranial trauma was also significant (0.88 times (0.83 to 0.94), p=0.001). A sensitivity analysis including only participants without amputation confirmed that this effect was not driven by the presence of amputees in the TBI group (B_TBI versus uninjured_=0.85 (0.82 to 0.90), p<0.001; B_polytrauma versus uninjured_=0.95 (0.93 to 0.98), p<0.001; B_TBI versus polytrauma_=0.90 (0.86 to 0.95), p<0.001).

As a global measure of function, employment status (ie, whether a participant was in paid work or training) was assessed at the time of the study visit. Only 76.5% of the TBI group were engaged in paid work or training, compared with 80.9% of the extracranial injury group and 96.6% of the unexposed group. On logistic regression accounting for age and rank, the odds of being out of work/training after TBI were 7.3 times greater (95% CI 3.5 to 16.0, p<0.001) than those in the unexposed group, although this was not significant on post hoc analysis comparing only TBI with extracranial injury.

### Chronic elevation in plasma GFAP after TBI

**Figure 3:**
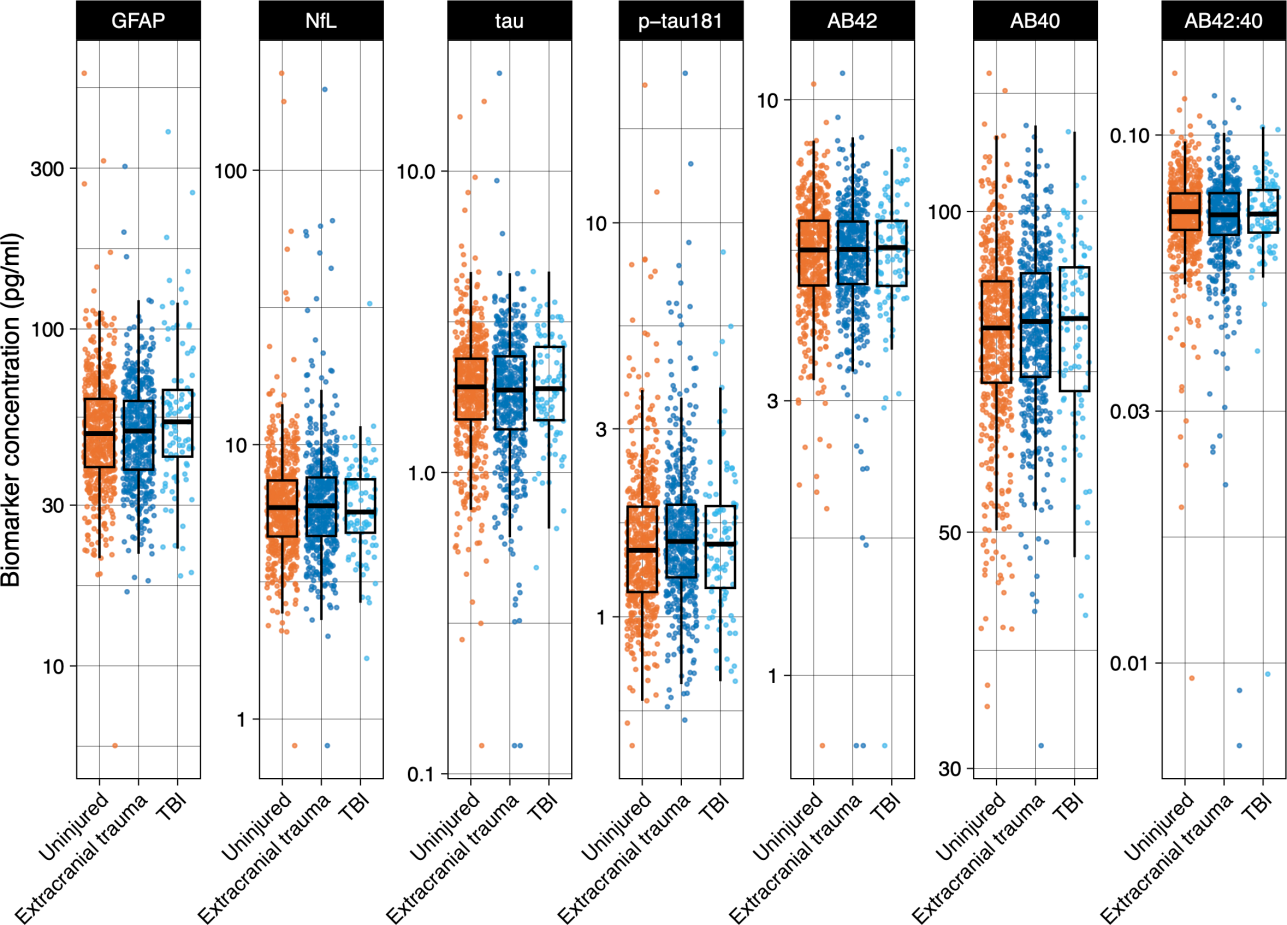
Plasma neurodegeneration biomarkers after traumatic brain injury (TBI). Box plots showing concentrations at baseline study visit of biomarkers related to TBI/neurodegeneration. Participants with no trauma shown in orange, extracranial injury in blue and TBI in cyan. Median is represented by the mid-horizontal line, with hinges showing the IQR. Whiskers show 1.5 times the IQR. Individual datapoints are plotted. Red indicates patient with extracranial injury, blue indicates patient with TBI. AB40, amyloid-β40; AB42, amyloid-β2; AB42:40, ratio of amyloid-β42 to 40; GFAP, glial fibrillar acidic protein; NfL, neurofilament light; p-tau_181_, tau phosphorylated at serine 181; tau, total tau.

Fluid biomarkers of TBI and neurodegeneration were quantified in 1122 participants, representing 98.0% of the total cohort (figure 3). Predefined protein biomarkers of interest were neuro-axonal marker NfL and astroglial marker GFAP, with exploratory analyses of Aβ40, Aβ42, p-tau_181_ and total tau (table 2). Plasma GFAP was significantly elevated after TBI, being 11% higher (95% CI 2% to 21%, geometric mean ratio, adjusted for age/rank, p=0.013) than unexposed participants. The elevation in plasma GFAP was TBI-specific, with no elevation in the extracranial injury group. GFAP after TBI was 13% higher (95% CI 3 to 23, p=0.007) than in participants with extracranial injury alone. There was no significant TBI-associated elevation in concentration of NfL, Aβ40, Aβ 42, Aβ42:40 ratio, p-tau_181_ or total tau (after adjusting for confounders age and rank) (online supplemental table 4).

**Table 2:** Plasma concentrations of neurodegeneration fluid biomarkers

	Trauma unexposed	Trauma exposed	
		Extracranial trauma	Traumatic brain injury
Biomarker data available, n (%)	558 (98.6)	469 (97.5)	95 (96.9)
GFAP, g.mean (g.SD), pg/mL	49.7 (1.48)	49.0 (1.43)	55.9 (1.68)
NfL, g.mean (g.SD), pg/mL	6.00 (1.59)	6.22 (1.67)	5.86 (1.49)
Tau, g.mean (g.SD), pg/mL	1.85 (1.6)	1.78 (1.64)	1.89 (1.51)
P-tau_181_, g.mean (g.SD), pg/mL	1.54 (1.53)	1.62 (1.55)	1.58 (1.56)
Aβ42, g.mean (g.SD), pg/mL	5.34 (1.26)	5.32 (1.29)	5.33 (1.33)
Aβ40, g.mean (g.SD), pg/mL	76.2 (1.22)	77.6 (1.2)	77.1 (1.22)
Aβ42:40 ratio, g.mean (g.SD), au	0.07 (1.23)	0.07 (1.28)	0.07 (1.29)

Aβ40amyloid-β40Aβ42, amyloid-β42GFAP, glial fibrillar acidic protein; g.meangeometric meang.SDgeometric standard deviationNfL, neurofilament light; p-tau_181_, tau phosphorylated at serine 181; tau, total tau

The result was robust to a sensitivity analysis in a subgroup of n=1081 participants with no history of other non-index (eg, prior or subsequent TBIs; see online supplemental table 1), with significantly higher plasma GFAP in participants with index TBI still evident (12% higher (95% CI 2 to 23), p=0.014).

### TBI severity and fluid biomarkers

The effect of TBI severity was assessed by comparing participants with moderate-to-severe TBI with those with mild-probable TBI. Plasma GFAP was 47% higher (95% CI 20% to 82%, p<0.001). There was no severity-associated change in concentrations of the other biomarkers (all analyses adjusted for confounders age and rank, see online supplemental table 5). A sensitivity analysis comparing only participants with moderate-to-severe TBI with unexposed participants was performed, given the possible influence of self-reported injuries in the mild TBI group. In this analysis, GFAP levels were significantly elevated, being 42% higher (95% CI 26 to 61, p<0.001).

### Individual-level elevations in plasma GFAP

Next, we investigated the associations of elevated plasma GFAP at the individual level using the 97.5th centile of the control (unexposed) distribution to define participants with ‘abnormally raised’ GFAP. This was found in 14 (2.5%) of unexposed participants, 6 (1.2%) with extracranial injury and 9 (9.2%) of participants with TBI, all of whom had moderate-to-severe TBI. Demographic/Clinical characteristics within the moderate-to-severe TBI group were compared. Participants with moderate-to-severe TBI who had elevated GFAP levels did not differ from those without elevated GFAP in terms of sex or rank. However, they typically had more severe injuries (NISS 34.00 (95% CI 25.00 to 36.00) vs 22.00 (12.50 to 28.00) in the uninjured group, p=0.039, W=203.5). Additionally, their injuries were more recent (time since injury 7.17 (95% CI 6.67 to 8.58) vs 9.33 (7.42 to 10.58) years, p=0.011, W=60.5).

### Time since injury

We explored the role of time since injury in the exposed group only. When time since injury was included in the model along with the interaction with TBI severity, a significant interaction was observed for the outcomes GFAP (p=0.009) and Aβ42 (p=0.029; see online supplemental table 6). There was no interaction of time since injury and group for AB40, the AB42:40 ratio, NfL, tau or p-tau_181_.

### Relationship between GFAP and clinical outcome measures in participants with TBI

The association of plasma GFAP and clinical outcomes was assessed in the TBI group, focused on this biomarker only given its elevation at the group level. On logistic regression assessing just the participants with TBI, a doubling of GFAP was associated with a 1.14-fold (95% CI 1.03 to 1.27, p<0.001) increase in the odds of being out of work or training. However, there was no significant correlation between plasma GFAP levels and depressive, anxiety or PTSD symptoms, or with 6 min walk distance (online supplemental figure 1).

## Discussion

A substantial proportion (~17%) of participants with major combat trauma in the ADVANCE cohort experienced a TBI, of which around a third were moderate-to-severe. Neurotrauma was associated with greater extents of affective symptoms and worse quality of life, motor and employment outcomes. Plasma GFAP, a marker of glial activation, was significantly elevated an average of 8 years after the traumatic injury and increased levels were associated with a reduced likelihood of working.

TBI is associated with a range of adverse clinical outcomes including cognitive problems, psychiatric symptoms and functional impairment, with accumulating evidence that these often extend into the chronic phase after injury. Such symptoms may also be present after injuries which are clinically classified as ‘mild’.^9^ Extending previous findings,^31^ we identified significantly higher extents of affective symptoms, including anxiety and depressive symptoms after TBI, as well as PTSD symptoms. Almost half the TBI-exposed cohort had at least mild depression/anxiety, with a quarter experiencing clinically significant PTSD symptoms. Patients with TBI were also significantly more likely to have motor impairments, and servicemen with TBI were more likely be out of work and report poor quality of life. A key strength of our study is the large extracranial (non-TBI injured) comparison group, which provided evidence that poor neuropsychiatric, motor and quality of life outcomes were specific to TBI, rather than being related to injury more generally.

Raised plasma GFAP has previously been described 5 years following human TBI,^17^ and our investigation extends this finding further into the chronic phase, suggesting ongoing astrocytic activation long after TBI. We found that GFAP was significantly raised in patient plasma after TBI compared with uninjured controls, as well as participants with extracranial trauma and that participants with moderate-to-severe TBI had higher GFAP than those with mild-probable injuries. Astrocytic activation is an early part of the neuroinflammatory injury response, characterised by expansion in cellular size, increased GFAP expression and astrocytic proliferation,^32^ with chronic astrogliosis a noted pathological feature of CTE.^33^ Disruption to the blood-brain barrier, of which GFAP-containing astroglial end-feet are an integral component,^34^ represents a further putative mechanism of raised plasma concentrations post-TBI.

The finding of raised GFAP this late postinjury is potentially of considerable mechanistic importance. In the context of AD, GFAP is more associated with amyloid-β than tau pathology.^35^ In familial AD, GFAP is one of the earliest plasma biomarkers to increase, before p-tau_181_ and NfL,^36^ with levels diverging between carriers and non-carriers around 16 years before estimated disease onset.^37^ In culture, astroglial activation promotes amyloid-β-driven tau phosphorylation,^38^ with experimental studies showing a reduced burden of tau pathology when activation is inhibited.^39^ Recent human work in sporadic late-onset AD recapitulates this, showing astrocyte reactivity status, evidenced by elevated plasma GFAP, is necessary for amyloid-β-dependent pathological tau phosphorylation, indicated by tau PET positivity.^40^

There is interest in the long-term trajectories of fluid biomarkers of injury and neurodegeneration, given increased prevalence of dementia post-TBI. An exploratory analysis of the interaction of sample time since injury and biomarker concentrations was performed, suggesting a TBI-associated reduction in GFAP, which may point to resolving rather than accelerating pathology and is in contrast to elevation of GFAP in AD.^41^ Individual-level analysis of plasma GFAP suggested that patients with more severe or more recent injuries were more likely to have significantly elevated levels. Conversely, there was a TBI-associated reduction in Aβ42, which is seen in the development of AD. Increased amyloid deposition has previously been shown on positron emission tomography in the chronic phase after moderate-to-severe TBI.^42^ However, the finding of reduced Aβ42 over time since injury has not been previously reported. Further work using longitudinal within-individual follow-up will be required to clarify the significance of these changes.

We found a significant relationship between plasma levels of GFAP in the chronic phase post-injury and employment outcomes, with higher GFAP associated with TBI-exposed participants being out of work and not in training. However, there were no correlations between biomarker concentrations and psychiatric symptom scores/walking speed, which may reflect underpowering for these exploratory analyses, with employment status in contrast capturing the end product of a broad range of impairments. There is limited research on fluid neurodegeneration biomarkers nearly a decade postinjury and their relation to functional status. One small study showed elevated plasma GFAP levels in a small number of patients late postinjury; however, unlike NfL, GFAP levels did not correlate with progressive functional decline.^19^

There are several potential limitations. TBI was ascertained using medical history and registry data relating to the index major combat trauma by which each participant was defined as ‘trauma exposed’ for the purposes of the ADVANCE study. It is possible that there were unrecognised previous injuries unaccounted for in this analysis: detailed ascertainment using the Ohio State questionnaire is currently being performed as the cohort undergoes follow-up assessment. A wide range of estimates of TBI prevalence have previously been reported in the Five Eyes countries. Here, TBI was a relatively common feature of combat trauma in nearly a fifth of the exposed group. However, by design, the study is not a representative sample of the UK Afghanistan experience, as it is enriched for traumatic injuries. It is therefore difficult to compare with other populations. As the cohort comprises male service personnel only, the generalisability of our findings in females will need to be assessed in future. Cognitive data were not available to relate to plasma biomarker concentrations but longitudinal follow-up including additional phenotyping is envisaged. In relation to statistical analysis, there was a limited amount of missing data, and a complete cases analysis approach was used throughout. We do not believe that this is likely to have influenced the core findings.

In conclusion, in the large military ADVANCE cohort of UK servicemen, we show poorer psychological, quality of life and functional outcomes after TBI, and plasma biomarker evidence of chronic astroglial activation 8 years postinjury. This indicates an ongoing process which may be indicative of a raised long-term neurodegeneration risk within a subset of the cohort. Future work within ADVANCE will incorporate the life-course TBI exposure and attempt to quantify the influence of repetitive head impacts, as well as the role of inflammatory mediators postinjury, such as via proteomic analysis.

## supplementary material

10.1136/jnnp-2024-333777 online supplemental file 1

## Data Availability

Data are available on reasonable request.
