# Supplementary material for: Poor long-term outcomes and abnormal neurodegeneration biomarkers after military traumatic brain injury: the ADVANCE study
Source: J Neurol Neurosurg Psychiatry. 2024 Oct 11;96(2):e333777. doi: 10.1136/jnnp-2024-333777 (PMC11877046; doi:10.1136/jnnp-2024-333777)
Supplement: online supplemental file 1 [file jnnp-96-2-s001.docx]

Supplementary Materials

**RESULTS**

**Supplementary Table 1. Psychiatric Comorbidity, Neurological Comorbidity and Non-index TBI Exposure**

|  | **Index Injury** | | | | | |
| --- | --- | --- | --- | --- | --- | --- |
|  | **All** | **Uninjured** | **Polytrauma** | **All TBI** | ***mild (probable)*** | ***moderate-severe*** |
| ***Psychiatric comorbidities*** |  |  |  |  |  |  |
| *Any psychiatric disorder* | 499 (43.6) | 174 (30.7) | 235 (48.9) | 90 (91.8) | 53 (101.9) | 37 (80.4) |
| PTSD | 198 (17.3) | 55 (9.7) | 106 (22) | 37 (37.8) | 24 (46.2) | 13 (28.3) |
| Depression | 153 (13.4) | 58 (10.2) | 71 (14.8) | 24 (24.5) | 14 (26.9) | 10 (21.7) |
| Anxiety / panic disorder | 91 (7.9) | 30 (5.3) | 40 (8.3) | 21 (21.4) | 11 (21.2) | 10 (21.7) |
| Other | 15 (1.3) | 7 (1.2) | 5 (1.0) | 1 (1.0) | 0 (0.0) | 1 (2.1) |
| Irritability/anger | 14 (1.2) | 6 (1.1) | 6 (1.2) | 2 (2) | 2 (3.8) | 0 (0.0) |
| Stress/stress reaction | 8 (0.7) | 5 (0.9) | 3 (0.6) | 0 (0.0) | 0 (0.0) | 0 (0.0) |
| Adjustment disorders | 7 (0.6) | 6 (1.1) | 0 (0.0) | 1 (1) | 0 (0.0) | 1 (2.2) |
| Deliberate self-harm | 4 (0.3) | 4 (0.7) | 0 (0.0) | 0 (0.0) | 0 (0.0) | 0 (0.0) |
| Sleep disorder | 4 (0.3) | 1 (0.2) | 2 (0.4) | 1 (1) | 1 (1.9) | 0 (0.0) |
| Attentional problems | 3 (0.3) | 0 (0.0) | 0 (0.0) | 3 (3.1) | 1 (1.9) | 2 (4.3) |
| Mood disorder | 2 (0.2) | 2 (0.4) | 0 (0.0) | 0 (0.0) | 0 (0.0) | 0 (0.0) |
| ***Neurological comorbidities*** |  |  |  |  |  |  |
| *Any (non-TBI) neurological disorder* | 46 (4) | 15 (2.7) | 12 (2.5) | 19 (19.4) | 7 (13.5) | 12 (26.1) |
| Seizure disorder | 23 (2) | 11 (1.9) | 6 (1.2) | 6 (6.1) | 1 (1.9) | 5 (10.9) |
| Cognitive difficulties | 5 (0.4) | 0 (0.0) | 3 (0.6) | 2 (2) | 1 (1.9) | 1 (2.2) |
| Heaadache disorder | 5 (0.4) | 1 (0.2) | 2 (0.4) | 2 (2) | 1 (1.9) | 1 (2.2) |
| Sensory disturbance | 4 (0.3) | 1 (0.2) | 0 (0.0) | 3 (3.1) | 3 (5.8) | 0 (0.0) |
| Vestibular disorder | 4 (0.3) | 1 (0.2) | 0 (0.0) | 3 (3.1) | 0 (0.0) | 3 (6.5) |
| Cerebrovascular disease | 1 (0.1) | 0 (0.0) | 0 (0.0) | 1 (1) | 0 (0.0) | 1 (2.2) |
| Cranial neuropathy | 1 (0.1) | 1 (0.2) | 0 (0.0) | 0 (0.0) | 0 (0.0) | 0 (0.0) |
| Motor impairment | 1 (0.1) | 0 (0.0) | 0 (0.0) | 1 (1) | 0 (0.0) | 1 (2.2) |
| Neuro-ophthalmological disorder | 1 (0.1) | 0 (0.0) | 0 (0.0) | 1 (1) | 1 (1.9) | 0 (0.0) |
| Peripheral nerve disease | 1 (0.1) | 0 (0.0) | 1 (0.2) | 0 (0.0) | 0 (0.0) | 0 (0.0) |
| CNS inflammatory disease | 0 (0.0) | 0 (0.0) | 0 (0.0) | 0 (0.0) | 0 (0.0) | 0 (0.0) |
| Dementia/neuro-degenerative disease | 0 (0.0) | 0 (0.0) | 0 (0.0) | 0 (0.0) | 0 (0.0) | 0 (0.0) |
| ***Comorbid TBI (non-index)*** |  |  |  |  |  |  |
| ≥ 1 mild probable, N (%) | 62 (5.4) | 29 (5.1) | 21 (4.4) | 12 (12.2) | 6 (11.5) | 6 (13.0) |
| =1 moderate-severe, N (%) | 2 (0.2) | 1 (0.2) | 1 (0.2) | 0 (0.0) | 0 (0.0) | 0 (0.0) |

*TBI: traumatic brain injury. Index TBI refers to the TBI the time of major trauma for which the participant, if in the exposed group in the ADVANCE cohort, was defined as ‘exposed’.*

**Supplementary Table 2. Outcomes after TBI**

|  | **Uninjured** | **Extracranial Trauma** | **TBI** | | |
| --- | --- | --- | --- | --- | --- |
|  | (n=566) | (n=481) | *All (n=98)* | *Mild-probable (N=52)* | *Moderate-severe (n=46)* |
| **PHQ9 (Depressive symptoms)** |  |  |  |  |  |
| Median score [Q25-Q75] | 2.0 [0.0, 7.0] | 4.0 [1.0, 8.0] | 5.0 [3.0, 12.8] | 6.0 [3.0, 13.0] | 4.0 [2.2, 11.8] |
| Abnormal (mild or above), N (%) | 208 (36.7%) | 202 (42.0%) | 55 (56.1%) | 33 (63.5%) | 22 (47.8%) |
| Unknown, N (%) | 1 (0.2%) | 2 (0.4%) | 0 (0.0%) | 0 (0.0%) | 0 (0.0%) |
| **GAD7 (Anxiety symptoms)** |  |  |  |  |  |
| Median score [Q25-Q75] | 2.0 [0.0, 5.0] | 3.0 [0.0, 7.0] | 5.0 [2.0, 11.0] | 6.0 [1.0, 11.0] | 5.0 [2.0, 11.8] |
| Abnormal (mild or above), N (%) | 164 (29.0%) | 179 (37.3%) | 54 (55.1%) | 29 (55.8%) | 25 (54.3%) |
| Unknown, N (%) | 1 (0.2%) | 1 (0.2%) | 0 (0.0%) | 0 (0.0%) | 0 (0.0%) |
| **PCL4 (PTSD symptoms)** |  |  |  |  |  |
| Median score [Q25-Q75] | 23.0 [18.0, 31.0] | 27.0 [20.0, 39.0] | 35.5 [24.5, 50.2] | 36.5 [23.0, 48.5] | 34.5 [25.0, 51.2] |
| Abnormal (score >49), N (%) | 52 (9.2%) | 64 (13.3%) | 25 (25.5%) | 13 (25.0) | 12 (26.0%) |
| Unknown, N (%) | 7 (1.2%) | 11 (2.3%) | 2 (2.0%) | 0 (0.0%) | 2 (4.3%) |
| **6 minute walk distance** |  |  |  |  |  |
| M, Median [Q25-Q75] | 630.0 [567.5, 690.0] | 580.0 [499.2, 659.8] | 548.0 [480.0, 631.2] | 575.0 [486.2, 639.2] | 543.5 [461.2, 599.8] |
| Unknown, N (%) | 5 (0.9%) | 27 (5.6%) | 6 (6.1) | 3 (5.4%) | 3 (%7.1) |
| **EQ5D5L total score (Quality of life)** |  |  |  |  |  |
| Median [Q25-Q75] | 6.0 [5.0, 7.0] | 7.0 [6.0, 9.0] | 8.0 [6.0, 10.0] | 8.0 [7.0, 10.0] | 7.5 [6.0, 10.8] |
| Unknown, N (%) | 7 (1.2%) | 11 (2.3%) | 5 (5.1%) | 2 (3.4%) | 3 (7.5%) |
| **In paid work/training** |  |  |  |  |  |
| N (%) | 547 (96.6%) | 389 (80.9%) | 75 (76.5%) | 43 (86.0) | 32 (71.1) |
| Unknown, N (%) | 6 (1.1%) | 29 (6.0%) | 3 (6.1%) | 2 (3.4%) | 1 (2.5%) |

*TBI: traumatic brain injury. GAD7:General anxiety disorders 7 questionnaire. PHQ9: Patient Health Questionnaire-9. PCL4- PTSD Checklist for DSM4 (PTSD – post traumatic stress disorder, DSM4 – diagnostic and statistical manual version 4). EQ5D5L – quality of life questionnaire.*

**Supplementary Table 3. Regression results – outcome measures and injury status**

|  | Regression model | | | | | | *Confounders included in model* |
| --- | --- | --- | --- | --- | --- | --- | --- |
|  | **TBI vs uninjured** | | **Extracranial injury vs uninjured** | | **TBI vs extracranial injury** | |  |
|  | Coefficient, 95% CI (exponentiated) | P | Coefficient, 95% CI (exponentiated) | P | Coefficient, 95% CI (exponentiated) | P |  |
| **6 minute walk** | 0.79 (0.74-0.84) | <0.001 | 0.89 (0.86-0.92) | <0.001 | 0.88 (0.83-0.94) | <0.001 | age, rank |
| **GAD7** | 1.65 (1.34-2.03) | <0.001 | 1.17 (1.04-1.32) | 0.008 | 1.40 (1.14-1.73) | 0.001 | age,rank * |
| **PHQ9** | 1.65 (1.33-2.03) | <0.001 | 1.18 (1.05-1.33) | 0.006 | 1.39 (1.13-1.72) | 0.002 | age,rank * |
| **PCL4** | 1.30 (1.19-1.41) | <0.001 | 1.11 (1.06-1.17) | <0.001 | 1.17 (1.07-1.27) | 0.001 | age,rank |
| **EQ5D5L** | 1.27 (1.19-1.36) | <0.001 | 1.16 (1.12-1.20) | <0.001 | 1.10 (1.03-1.18) | 0.004 | age,rank |
|  | OR, 95% CI | P | OR, 95% CI | P | OR, 95% CI | P |  |
| **Employment (not in work/training)** | 7.33 (3.45-16.0) | <0.001 | 5.38 (3.00-10.4) | <0.001 | 1.36 (0.74-2.42) | 0.0302 | age,rank |

*Exponentiated coefficient = geometric mean ratio. GAD7:General anxiety disorders 7 questionnaire. PHQ9: Patient Health Questionnaire-9. PCL4- PTSD Checklist for DSM4. EQ5D5L – quality of life questionnaire. *Constant of 1 added to outcome measure to facilitate log transformation due to scores of zero.*

**Supplementary Table 4. Regression results – biomarkers and injury status**

|  | Regression model | | | | | | *Confounders included in model* |
| --- | --- | --- | --- | --- | --- | --- | --- |
|  | **TBI vs uninjured** | | **Extracranial injury vs uninjured** | | **TBI vs extracranial injury** | |  |
|  | Coefficient, 95% CI (exponentiated) | P | Coefficient, 95% CI (exponentiated) | P | Coefficient, 95% CI (exponentiated) | P |  |
| **GFAP** | 1.11 (1.02-1.21) | 0.013 | 0.99 (0.94-1.04) | 0.71 | 1.13 (1.03-1.23) | 0.007 | age, rank |
| **NfL** | 0.95 (0.86-1.06) | 0.369 | 1.06 (1-1.12) | 0.067 | 0.904 (0.82-1) | 0.053 | age, rank |
| **Tau** | 1.00 (0.90-1.11) | 0.961 | 0.95 (0.89-1.01) | 0.092 | 1.06 (0.95-1.17) | 0.315 | age, rank |
| **P-tau_181_** | 1.03 (0.94-1.14) | 0.51 | 1.06 (1.01-1.12) | 0.031 | 0.97 (0.88-1.07) | 0.572 | age, rank |
| **AB40** | 1.01 (0.97-1.05) | 0.719 | 1.02 (0.99-1.04) | 0.178 | 0.99 (0.95-1.03) | 0.685 | age, rank |
| **AB42** | 1.00 (0.95-1.06) | 0.861 | 1 (0.97-1.03) | 0.992 | 1.01 (0.95-1.06) | 0.857 | age, rank |
| **AB42:40** | 1.00 (0.95-1.05) | 0.910 | 0.98 (0.96-1.01) | 0.250 | 1.01 (0.96-1.07) | 0.590 | age, rank |

*TBI: traumatic brain injury. GFAP: glial fibrillary acidic protein. NfL: neurofilament light. Tau: total tau. P-tau_181_: tau phosphorylated at serine 181. Ab40: amyloid beta 40. AB42: amyloid beta 42.*

**Supplementary Table 5. Regression results – biomarkers and TBI severity model**

|  | **Moderate-severe TBI vs mild probable** | | *Confounders in model* |
| --- | --- | --- | --- |
|  | Coefficient, 95% CI (exponentiated) | P |  |
| **GFAP** | 1.37 (1.11-1.69) | 0.004 | age, rank |
| **NfL** | 1.15 (0.97-1.36) | 0.106 | age, rank |
| **Tau** | 1.03 (0.86-1.23) | 0.730 | age, rank |
| **P-tau_181_** | 1.01 (0.84-1.22) | 0.903 | age, rank |
| **AB40** | 1.02 (0.94-1.11) | 0.659 | age, rank |
| **AB42** | 0.97 (0.85-1.09) | 0.573 | age, rank |
| **AB42:40** | 0.95 (0.85-1.06) | 0.326 | age,rank |

*TBI: traumatic brain injury. GFAP: glial fibrillary acidic protein. NfL: neurofilament light. Tau: total tau. P-tau_181_: tau phosphorylated at serine 181. AB40: amyloid beta 40. AB42: amyloid beta 42. AB42:40: amyloid beta 42:40 ratio*

**Supplementary Table 6. Regression results – time since injury and biomarker concentrations**

|  | **Interaction Effect** | | **Main Effects** | | | | *Confounders in model* |
| --- | --- | --- | --- | --- | --- | --- | --- |
|  | Time since injury and group | | Time since injury | | Group (TBI, vs extracranial injury) | |  |
|  | Coefficient, 95% CI (exponentiated) | P | Coefficient, 95% CI (exponentiated) | P | Coefficient, 95% CI (exponentiated) | P |  |
| **GFAP** | 0.95 (0.91-0.99) | 0.009 | 1.00 (0.098-1.02) | 0.889 | 1.81 (1.26-2.60) | 0.001 | age, rank |
| **NfL** | 0.96 (0.91-1.01) | 0.083 | 1.00 (0.98-1.02) | 0.976 | 1.34 (0.85-2.10) | 0.206 | age, rank |
| **Tau** | 1.00 (0.95-1.05) | 0.973 | 1.00 (0.98-1.02) | 0.848 | 1.04 (0.66-1.65) | 0.850 | age, rank |
| **P-tau_181_** | 0.96 (0.92-1.01) | 0.113 | 0.99 (0.97-1.01) | 0.212 | 1.35 (0.89-2.04) | 0.162 | age, rank |
| **AB40** | 0.99 (0.97-1.01) | 0.478 | 0.99 (0.99-1.00) | 0.091 | 1.05 (0.89-1.25) | 0.544 | age, rank |
| **AB42** | 0.97 (0.94-1.00) | 0.029 | 0.99 (0.98-1.00) | 0.097 | 1.31 (1.03-1.68) | 0.029 | age, rank |
| **AB42:40** | 0.98 (0.95-1.00) | 0.077 | 1.00 (0.99-1.01) | 0.626 | 1.25 (0.99-1.57) | 0.065 | age,rank |

*TBI: traumatic brain injury. GFAP: glial fibrillary acidic protein. NfL: neurofilament light. Tau: total tau. P-tau_181_: tau phosphorylated at serine 181. Ab40: amyloid beta 40. AB42: amyloid beta 42. AB42:40 ratio of amyloid beta 42 to 40.*

**Supplementary Figure 1. Correlations of plasma GFAP and Outcome Measures**

*Pearson’s correlation’s between outcome measures for anxiety (GAD7), PTSD symptoms (PCL4), depressive symptoms (PHQ9) and six minute walk distance shown. GAD7:General anxiety disorders 7 questionnaire. PHQ9: Patient Health Questionnaire-9. PCL4- PTSD Checklist for DSM4. EQ5D5L – quality of life questionnaire.*
